# Neurophysiological Biomarkers of Persistent Post-concussive Symptoms: A Scoping Review

**DOI:** 10.3389/fneur.2021.687197

**Published:** 2021-09-09

**Authors:** Sepehr Mortaheb, Maria Maddalena Filippini, Jean-François Kaux, Jitka Annen, Nicolas Lejeune, Géraldine Martens, Maria Antonia Fuentes Calderón, Steven Laureys, Aurore Thibaut

**Affiliations:** ^1^Coma Science Group, GIGA-Consciousness, University of Liège, Liège, Belgium; ^2^Brain Clinic, University Hospital of Liège, Liège, Belgium; ^3^Physiology of Cognition Lab., GIGA-Consciousness, University of Liège, Liège, Belgium; ^4^Neuromotor and Rehabilitation Department, Azienda Unita Sanitaria Locale-Istituto di Ricovero e Cura a Carattere Scientifico (USL-IRCSS) di Reggio Emilia, Reggio Emilia, Italy; ^5^Physical Medicine and Sport Traumatology Department, Sports, FIFA Medical Centre of Excellence, IOC Research Centre for Prevention of Injury and Protection of Athletes Health, FIMS Collaborative Centre of Sport Medicine, University and University Hospital of Liège, Liège, Belgium; ^6^Institute of NeuroScience, University of Louvain, Brussels, Belgium; ^7^Neurorehabilitation (NEURORHB) Servicio de Neurorrehabilitación de Hospitales Vithas, Valencia, Spain

**Keywords:** neuroimaging, persistent post-concussive symptoms, diffusion weighted imaging, functional MRI, electroencephalography, mTBI

## Abstract

**Background and Objectives:** Persistent post-concussive symptoms (PCS) consist of neurologic and psychological complaints persisting after a mild traumatic brain injury (mTBI). It affects up to 50% of mTBI patients, may cause long-term disability, and reduce patients' quality of life. The aim of this review was to examine the possible use of different neuroimaging modalities in PCS.

**Methods:** Articles from Pubmed database were screened to extract studies that investigated the relationship between any neuroimaging features and symptoms of PCS. Descriptive statistics were applied to report the results.

**Results:** A total of 80 out of 939 papers were included in the final review. Ten examined conventional MRI (30% positive finding), 24 examined diffusion weighted imaging (54.17% positive finding), 23 examined functional MRI (82.61% positive finding), nine examined electro(magneto)encephalography (77.78% positive finding), and 14 examined other techniques (71% positive finding).

**Conclusion:** MRI was the most widely used technique, while functional techniques seem to be the most sensitive tools to evaluate PCS. The common functional patterns associated with symptoms of PCS were a decreased anti-correlation between the default mode network and the task positive network and reduced brain activity in specific areas (most often in the prefrontal cortex).

**Significance:** Our findings highlight the importance to use functional approaches which demonstrated a functional alteration in brain connectivity and activity in most studies assessing PCS.

## Introduction

Emerging evidence shows that the long-term consequences of concussion—also known as mild traumatic brain injury (mTBI)—can be broad and last long (1). Those consequences can last for a few minutes (e.g., dizziness and loss of consciousness), for a few hours (e.g., post-traumatic amnesia, headaches, and attentional and/or sensitivity issues), or for weeks or months (e.g., fatigue, irritability, anxiety, and insomnia), which are known as persistent post-concussive symptoms (PCS) and can have an important socio-economic impact (2). Up to 50% of patients who had a concussion suffer from PCS at 3 months post injury (3). One study highlighted that 27% of patients who had a concussion could not return to their previous work 12 months post-injury; furthermore, among the patients who return to their previous work, 84% still reported PCS-related complaints (4). These authors also showed that, besides demographics predictors (e.g., age and education) and injury characteristics (e.g., cause and severity of injury), the indicators of psychological distress and employment were of influence on work resumption. Proper diagnosis of a concussion is the first step to an accurate disease management which can further enable the re-employment of subjects and can reduce economic burden on the society. Lack of knowledge (e.g., inaccurate belief that the concussion is systematically linked to loss of consciousness) and unfamiliarity with common guidelines in sports teams (despite the increasing interest of international federations in establishing new guidelines and regulations) are also a serious issue which limit proper diagnosis and the appropriate management of patients who suffered from mTBI (5, 6).

The heterogeneity of injury and symptoms, the current limitations in the sensitivity of imaging, and the lack of sensitivity of classical biological markers represent important challenges for the development of diagnostic tools and biomarkers of good and poor recovery. Acute conventional computed tomography (CT) findings do not correlate with long-term outcomes such as PCS (7, 8). An extensive meta-analysis suggests that CT should only be performed under certain circumstances, such that when patients are at risk of severe intracranial injuries and present specific symptoms (e.g., loss of consciousness for more than 5 min, declining in neurological status, or having seizures among others) (9). Besides CT, other techniques offer alternatives to study brain structural and functional integrity, such as MRI [conventional MRI, functional MRI (fMRI), diffusion-weighted imaging (DWI), magnetic resonance spectroscopy (MRS)], single-photon emission computed tomography (SPECT), positron emission tomography (PET), magnetoencephalography (MEG), electroencephalography (EEG), or functional near-infrared spectroscopy (fNIRS). Neuroimaging techniques have been shown to be sensitive biomarkers for an accurate diagnosis and prognosis of PCS. Importantly, damage to the structural integrity (e.g., alterations of anisotropy and diffusion measures of the white matter) and disrupted functional network communication (e.g., alterations in resting-state functional network connectivity or in regional activations while performing a cognitive task) in the acute and subacute phases of mTBI are the main factors which lead to PCS (10). While these biomarkers are sufficiently sensitive to detect concussion-related symptoms, there are also some inconsistencies across studies about the way these markers are related to the concussion (11). As a result, more studies and reviews are needed for a comprehensive understanding of PCS and how neuroimaging biomarkers can help to diagnose and prognose such syndrome.

Taking all these into account, our review aims to address which neuroimaging features of mTBI are relevant to the clinical expression of PCS and could therefore potentially be used as biomarkers for PCS diagnosis and/or prognosis whether cross-sectionally or longitudinally.

## Materials and Methods

A search on PubMed database was performed on June 13, 2018 and updated on May 14, 2020, which included the following terms: mTBI, mild traumatic brain injury, post-concussive syndrome, post-concussion syndrome, and neuroimaging, magnetic resonance imaging, functional magnetic resonance imaging, diffusion tensor imaging, diffusion spectrum imaging, diffusion-weighted imaging, susceptibility-weighted imaging, diffusion kurtosis imaging, positron emission tomography, computed tomography, single-photon emission computed tomography, magnetoencephalography, electroencephalography, near-infrared spectroscopy, functional near-infrared spectroscopy, resting state, and functional connectivity.

The inclusion criteria were as follows: (1) articles published in the last 10 years (from the time of database search, which leads to articles published from 2008), (2) in humans (adults: 16+ years old), (3) on post-concussion syndrome, (4) patients had to be assessed with one of the following techniques: CT, MRI, PET, SPECT, MEG, NIRS, or EEG, and (5) published in English.

The exclusion criteria were as follows: (1) articles including <12 subjects to exclude case studies and pilot studies [based on the criteria discussed in Julious (12)] and (2) reviews, opinion papers, or case reports.

Our outcome measures involved the association between neuroimaging and/or neurophysiological biomarkers and the presence or severity of PCS symptoms as measured cross-sectionally or longitudinally with the following scales (non-exhaustive list): Post-concussion Symptom Scale, Post-concussive Symptom Questionnaire, PCS-19, PCS-Negative Impression Management Scale, Sport Concussion Assessment Tool (all versions), International Classification of Diseases−10th edition Diagnostic Criteria for Post-concussion Syndrome, Diagnostic and Statistical Manual for Mental Disorders—IVth edition Diagnostic Criteria for Post-concussional Disorder, Rivermead Post-concussion Symptoms Questionnaire, British Columbia Post-concussion Symptom Inventory, Neurobehavioral Symptom Inventory, and Immediate Post-concussion Assessment and Cognitive Testing. In order to prevent differences in brain development and because some of the mentioned behavioral scales are not validated for adolescents, we limited our review to the studies with subjects older than 16 years old.

Two independent blinded reviewers (SM and MF) first reviewed the abstracts for inclusion using the Rayyan software (13). In case of disagreement, a third reviewer (AT) was involved in the final decision. Descriptive statistics were used to report the results. In this review, “positive results” refer to the studies for which we found a statistically significant association between at least one neuroimaging marker and PCS.

## Results

**Figure 1:**
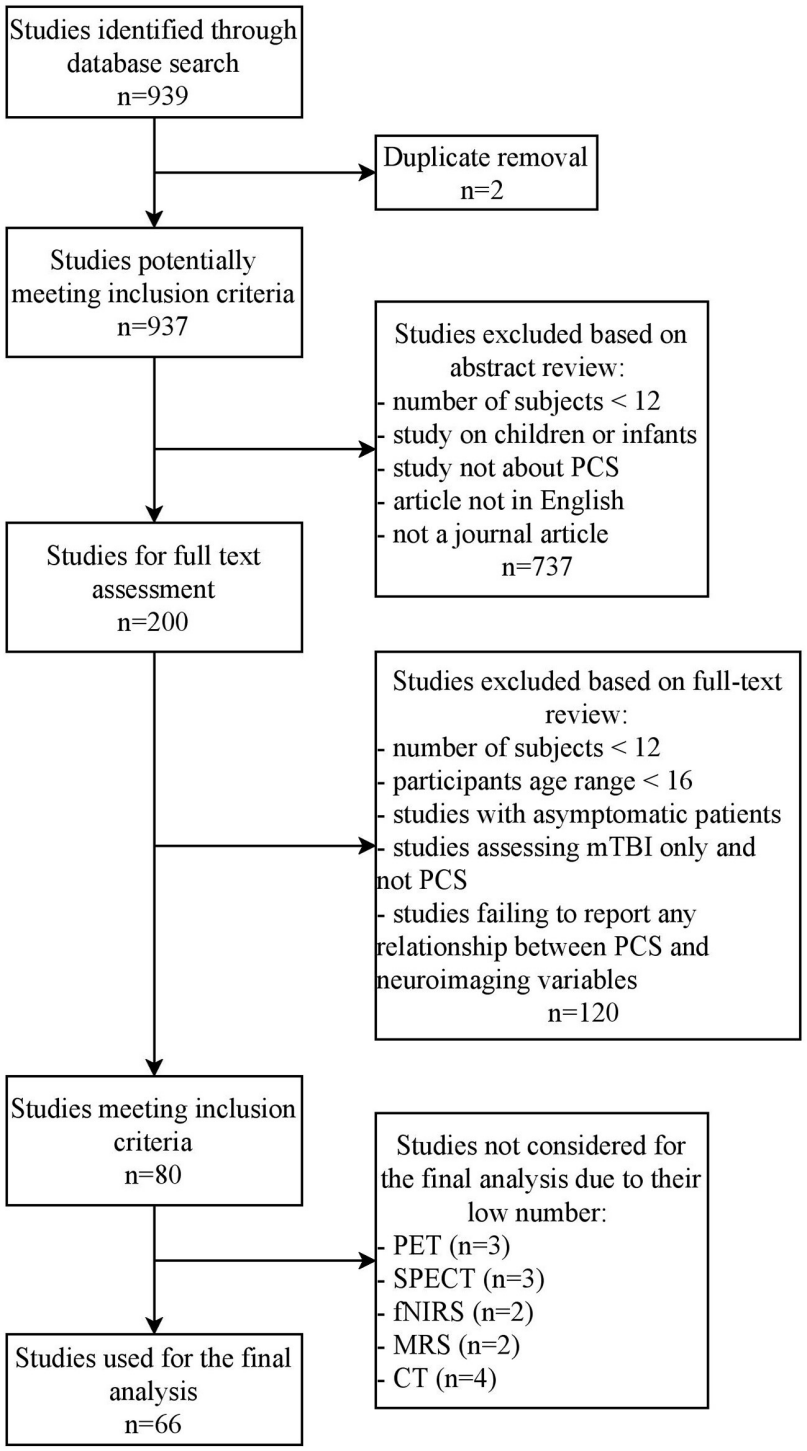
PRISMA flowchart. A search over the Pubmed database yielded 939 studies, after which they were screened in the first round based on their abstracts and in the second round based on the main text of the remaining studies (PET, Positron Emission Tomography; SPECT, Single Photon Emission Computed Tomography; fNIRS, Functional Near Infrared Spectroscopy; MRS, Magnetic Resonance Spectroscopy; CT, Computed Tomography).

The search yielded 939 articles, from which 859 were excluded and 80 articles were kept for a final review (see Figure 1). The imaging techniques used in these 80 studies were conventional MRI (10 papers), DWI (24 papers), functional MRI (23 papers), EEG/MEG (nine papers), PET (three papers), SPECT (three papers), fNIRS (two papers), MRS (two papers), and CT (four papers).

**Figure 2:**
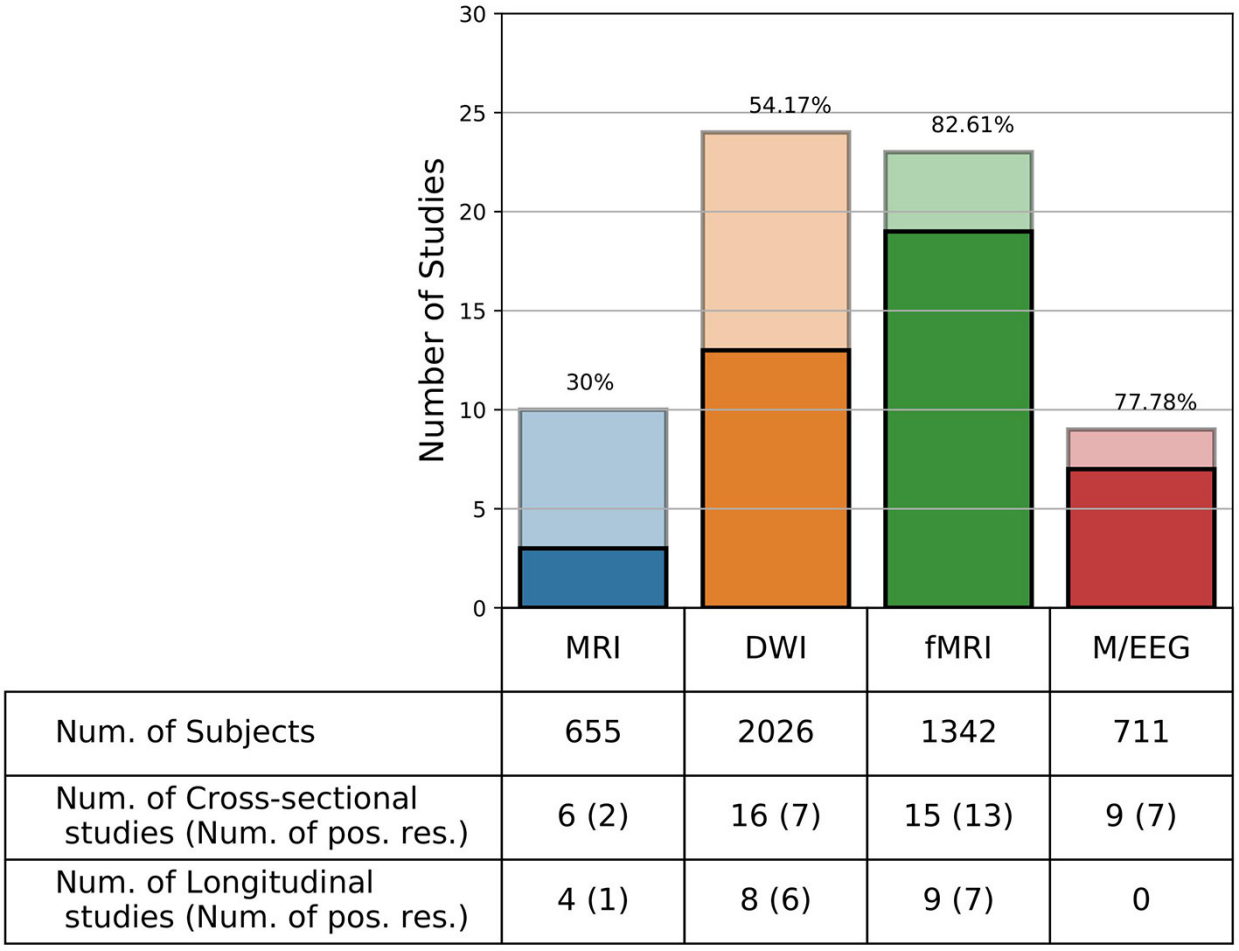
Number of studies per imaging technique. Number of studies (Y-axis) per imaging technique (X-axis). Functional MRI has the highest number of experiments with positive results (i.e., studies which found an association between neuroimaging features and post-concussive symptoms). Darker colors show the number of studies with positive results, while lighter colors show the studies without significant findings. The number above each bar represent the percentage of studies with positive results.

We here present the results for conventional MRI, DWI, fMRI, and EEG/MEG (66 papers; see Figure 2 and Table 1; for the definitions of some specific outcome measures mentioned in this table, see Table 2). For the other techniques, only a couple of articles were found (*n* = 14), which limits the generalizability of the results.

**Table 1:** Studies included in the final review, sorted by neuroimaging technique.

**References**	**N_Total (N_PCS)**	**Age of the patients (years)**	**Time post-injury**	**Positive/** **negative results**	**Source of injury**	**Study design**	**Main markers**
**Conventional MRI**							
Datta et al. (14)	20 (20)	23–70 (mean = 35)	3–84 m	Negative	Accident Falls Assault	Cross-sectional	MRI lesions
Sigurdardottir et al. (15)	115 (25)	35.9 ± 11.4	T1: 3 m T2: 12 m	Negative	Accident Fall Assault Sport-related	Longitudinal	Intracranial pathology
Zhou et al. (16)	50 (n.a.)	18–56 (34 ± 11.5)	T1: 3–53 d (mean = 23) T2: 1 y	Positive	Accident Falls Assault Sport-related	Longitudinal	Global and local atrophies
Lannsjö et al. (17)	19 (n.a.)	17–63 (mean = 34)	T1: 2–3 d T2: 3–7 m	Negative	Accident Fall	Longitudinal	Volume changes
Killgore et al. (18)	38 (n.a.)	20–45 (23.38 ± 5.23)	2 w, 1 m, 3 m, 6 m, 1 y	Positive	n.a.	Cross-sectional	GM volume alterations
Clark et al. (19)	68 (46)	30.97 ± 7.5	6–121 m	Negative	n.a.	Cross-sectional	WM hyperintensities
Hellstrøm et al. (20)	62 (n.a.)	16–65	T1: 30.42 d T2: 1 y	Negative	Accident Fall Violence	Longitudinal	Volume changes
De Haan et al. (21)	127 (n.a.)	16–76 (39 ± 15)	24 ± 14 w	Negative	n.a.	Cross-sectional	Microhaemorrhage
Chai et al. (22)	80 (n.a.)	19–58 (35.38 ± 10.6)	12–360 h (70.73 ± 73.4)	Positive	Accident Fall Assault Falling objects Blast	Cross-sectional	Cerebral venous oxygen saturation alterations
Yoo et al. (23)	76 (44)	23–81 (mean = 47)	4 m	Negative	n.a.	Cross-sectional	Blood–brain barrier disruption
**Diffusion-weighted imaging**							
Smits et al. (24)	32 (n.a.)	26 ± 7.4	18–40 d	Positive	n.a	Cross-sectional	Anisotropy and diffusion measures
Messé et al. (25)	46 (12)	30.6 ± 8.6	T1: 7–28 d T2: 3–4 m	Positive	Accident Fall Aggression	Longitudinal	Anisotropy and diffusion measures
Messé et al. (26)	93 (22)	35.5 ± 11	T1: 8–21 d T2: 6 m	Positive	n.a.	Longitudinal	Anisotropy and diffusion measures
Lange et al. (27)	94 (21)	30.8 ± 9.9	47 ± 6,3 d	Negative	Accident Non-accident	Cross-sectional	Anisotropy and diffusion measures
Yeh et al. (28)	51 (n.a.)	28.1 ± 5.6	405 ± 548.8 d	Positive	Accident Fall Blast	Cross-sectional	Anisotropy and diffusion measures
Ilvesmäki et al. (29)	115 (32)	37.2 ± 12	48.1 ± 45.4 h	Negative	n.a.	Cross-sectional	Anisotropy and diffusion measures
Wäljas et al. (30)	72 (11)	36.4 ± 12.4	16–60 d	Negative	n.a.	Cross-sectional	Anisotropy and diffusion measures
Petrie et al. (31)	52 (n.a.)	23–60(31.6 ± 9.2)	1.2–7.1 y	Negative	Blast	Cross-sectional	Anisotropy and diffusion measures
Panenka et al. (32)	94 (n.a.)	30.6 ± 8.67	42–56 d	Negative	Accident Fall Assault Sport-related	Cross-sectional	Microstructural changes
Davenport et al. (33)	125 (n.a.)	22–59(32.8 ± 8.5)	2–5 y	Negative	Blast	Cross-sectional	WM integrity measures
Lange et al. (34)	108 (20)	34.1 ± 11	46.77 ± 6.2 d	Positive	Accident Non-accident	Cross-sectional	Anisotropy and diffusion measures
Wäljas et al. (35)	186 (124)	16–64(37.8 ± 13.5)	8–38 d	Negative	Accident Fall Assault Sport-related	Cross-sectional	Microstructural changes
Delano-Wood et al. (36)	58 (n.a.)	33.37 ± 6.4	16–259 m	Positive	Blast Blunt	Cross-sectional	Anisotropy and diffusion measures
Miller et al. (37)	90 (n.a.)	28.5 ± 5.89	50 m	Positive	Blast	Cross-sectional	Anisotropy and diffusion measures
Meier et al. (38)	86 (n.a.)	20.12 ± 1.4	T1: 1.64 d T2: 8.33 d T3: 32.15 d	Negative	Sport-related	Longitudinal	Anisotropy and diffusion measures
Lancaster et al. (39)	52 (n.a.)	17.6 ± 1.5	T1: 14-24 h T2: 8 d	Positive	Sport-related	Longitudinal	Anisotropy, diffusion, and kurtosis measures
Astafiev et al. (40)	42 (n.a.)	39.8 ± 11.3	n.a.	Negative	Accident Fall Sport related	Cross-sectional	Anisotropy and diffusion measures
Thomas et al. (41)	36 (20)	18–57 (mean = 30.6)	T1: 46.5 ± 22 h T2: 1 w	Positive	n.a.	Longitudinal	Anisotropy and diffusion measures
Yeh et al. (42)	242 (202)	20–50 (31.9 ± 7.3)	576 ± 238.9 d	Positive	Blast	Cross-sectional	Anisotropy and diffusion measures, Microstructural changes
Næss-Schmidt et al. (43)	54 (n.a.)	27.6 ± 6.4	T1: 14 d T2: 3 m	Negative	Accident Fall Assault	Longitudinal	Kurtosis measures
Rangaprakash (44)	87 (42)	33.7 ± 6.8	n.a.	Positive	n.a.	Cross-sectional	Structural connectivity
Klimova et al. (45)	110 (n.a.)	43.4 ± 10.2	125.6 ± 121.4 m	Negative	Accident Fall Assault	Cross-sectional	Anisotropy and diffusion measures
Lancaster et al. (46)	37 (n.a.)	17.5 ± 1.7	T1: 14–24 h T2: 7–11 d T3: 151–204 d	Positive	Sport-related	Longitudinal	Anisotropy and diffusion measures
Yin et al. (47)	64 (n.a.)	37.7 ± 13.6	T1: 0–5 d T2: 27–35 d T3: 85–105 d	Positive	n.a.	Longitudinal	Anisotropy and diffusion measures, WM integrity measures
**Functional MRI**							
Smits et al. (48)	33 (21)	25.9 ± 7.8	30 d	Positive	n.a.	Cross-sectional	Functional activation during task
Tang et al. (49)	41 (n.a.)	22–62 (mean = 37.3)	22 d	Positive	n.a.	Cross-sectional	Resting-state functional connectivity
Gosselin et al. (50)	37 (n.a.)	31.9 ± 12.7	5.7 m	Positive	Recreational accident Work-related accident Vehicular accident	Cross-sectional	Functional activation during task
Stevens et al. (51)	60 (n.a.)	18–55 (31.7 ± 13.9)	60.9 ± 35.77 d	Positive	n.a.	Cross-sectional	Resting-state functional connectivity
Terry et al. (52)	40 (9)	20.3 ± 1.17	596.17 d	Negative	Sport-related	Cross-sectional	Functional activation during task
Zhou et al. (53)	41 (n.a.)	37.8 ± 12.9	22 d	Positive	n.a.	Cross-sectional	Resting-state functional connectivity
Messé et al. (54)	89 (17)	34.9 ± 11.5	T1: 1–3 w T2: 6 m	Positive	Accident Fall Aggression Work-related Sport-related	Longitudinal	Resting-state functional network graph metrics
Mutch et al. (55)	17 (12)	31.67 ± 8.79	n.a.	Positive	Accident Fall Sport-related Others	Cross-sectional	Regional cerebrovascular flow alterations
Sours et al. (56)	63 (15)	41.7 ± 17.1	T1: 6 d T2: 6 m	Positive	n.a.	Cross-sectional, longitudinal	Resting-state functional connectivity
Sours et al. (57)	56 (12)	38.9 ± 15.9	T1: 7 d T2: 1 m T3: 6 m	Negative	Accident Fall Assault Sport-related	Longitudinal	Resting-state network cerebral blood flow
Sours et al. (58)	112 (n.a.)	44 ± 17	6 d	Positive	Accident Fall Assault Sport-related Blunt	Cross-sectional	Resting-state functional connectivity
Iraji et al. (59)	28 (n.a.)	38 ± 17	0.54 d	Negative	Accident Fall Assault Sport-related	Cross-sectional	Resting-state functional connectivity
Koski et al. (60)	15 (12)	20–60 (34.3 ± 10.8)	T1: n.a. T2: 2 w T3: 3 m	Positive	n.a.	Longitudinal	Functional activation during task
Astafiev et al. (40)	42 (n.a.)	39.8 ± 11.3	n.a.	Positive	Accident Fall Sport-related	Cross-sectional	Functional activation during task, Resting-state functional connectivity
Van der Horn et al. (61)	72 (32)	19–64 (mean = 37.8)	30.42 d	Positive	Accident Fall Assault Sport-related	Cross-sectional	Functional activation during task, Resting-state functional connectivity
Meier et al. (38)	94 (n.a.)	20.29 ± 1.31	T1: 1.74 d T2: 1 w T3: 1 m	Negative	Sport-related	Longitudinal	Resting-state functional connectivity
Banks et al. (62)	24 (n.a.)	39.3 ± 14	T1: 42 d T2: 4 m	Positive	Accident Fall	Longitudinal	Resting-state functional connectivity
Palacios et al. (63)	122 (n.a.)	32.36 ± 10.7	T1: 11.2 d T2: 6 m	Positive	n.a.	Cross-sectional	Resting-state functional connectivity
Zhou et al. (64)	75 (n.a.)	35 ± 13	22 d	Positive	n.a.	Cross-sectional	Resting-state functional network graph metrics
Kaushal et al. (65)	122 (62)	18.9 ± 1.84	T1: 1.35 d T2: 8.2 d T3: 15.42 d T4: 45.56 d	Positive	Sport-related	Longitudinal	Resting-state functional connectivity
Chong et al. (66)	30 (15)	39.1 ± 10.1	T1: 1 m T2: 5 m	Positive	Accident Fall Sport-related	Longitudinal	Resting-state functional connectivity
Hou et al. (67)	77 (24)	41.7 ± 17.3	7 d	Positive	Accident Fall Assault Sport-related Blunt	Cross-sectional	Resting-state functional network graph metrics
Manning et al. (68)	52 (21)	18–22 (20.1 ± 1)	T1: 3 d T2: 3 m T3: 6 m	Positive	Sport-related	Longitudinal	Resting-state structure-function connectivity
**EEG/MEG**							
Gosselin et al. (50)	37 (n.a.)	31.9 ± 12.7	5.7 m	Negative	Recreational accident Work-related accident Vehicular accident	Cross-sectional	ERP component amplitude
Gosselin et al. (69)	84 (n.a.)	30.3 ± 11.1	7.6 m	Positive	Accident Assault Sport-related Work-related	Cross-sectional	ERP component amplitude
Larson et al. (70)	82 (n.a.)	21.6 ± 2.4	212.92 d	Negative	Accident Fall Sport-related	Cross-sectional	ERP component amplitude
Huang et al. (71)	163 (84)	29.4 ± 8.5	264.63 d	Positive	Blast Non-blast	Cross-sectional	MEG slow-wave
Swan et al. (72)	64 (31)	26.6 ± 6.1	97.4 d	Positive	Accident Fall Assault Sport-related	Cross-sectional	MEG slow-wave
Vakorin et al. (73)	41 (n.a.)	21–44 (31 ± 7)	32 d	Positive	n.a.	Cross-sectional	EEG resting-state phase synchrony
Huang et al. (74)	48 (26)	28.41 ± 4.5	507.35 d	Positive	Combat-related	Cross-sectional	MEG resting-state functional connectivity
Lewine et al. (75)	153 (71)	21–53 (mean = 33)	>5 m	Positive	Military-related	Cross-sectional	EEG power and coherency
Ruiter et al. (76)	39 (19)	45–66 (mean = 57.6)	28.11 y	Positive	sport related	Cross-sectional	ERP component amplitude

*DWI, diffusion-weighted imaging; EEG, electroencephalography; ERP, event-related potentials; GM, gray matter; ICB, intracranial bleeds; MEG, magnetoencephalography; N_Total, total number of participants in each study (including controls); N_PCS, number of patients diagnosed as PCS; n.a., not available; T1, baseline timepoint; Tn, follow-up timepoints; h, hour(s); d, day(s); w, week(s); m, month(s); y, year(s); WM, white matter*.

**Table 2:** Definition of specific outcome measures in the reviewed papers.

**Explanation**	**Outcome measure**	**Outcome measure definition**
**Diffusion-weighted imaging parameters**		
In diffusion tensor imaging, we can model water molecule diffusion by tensors. The three main eigenvalues and eigenvectors of tensor representation can give information about the main diffusion direction. In the following definitions, consider λ_1_ as the maximum eigenvalue (main diffusion direction) and λ_2_ and λ_3_ as the two shortest eigenvalues (perpendicular to the main diffusion direction).	Axial diffusivity (AD)	AD = λ_1_ It is only sensitive to diffusion in the longest eigenvalue direction. Highly organized structures like white matter pathways and large open cavities like ventricles have generally high levels of diffusion and are sensitive to this measure.
	Radial diffusivity (RD)	RD = (λ_2_ + λ_3_)/2 It represents the two shortest eigenvalues and shows low values in highly organized and dense structures like white matter pathways, intermediate values in gray matter, and high values in regions with cerebral spinal fluid (CSF)
	Mean diffusivity (MD)	MD = (λ_1_ + λ_2_ + λ_3_)/3. It is specifically sensitive to CSF, which has high values of average diffusion.
	Fractional anisotropy	It provides the relative difference between the largest eigenvalue as compared to the others; it quantifies the fraction of diffusion that is anisotropic. This leads to selective high values in white matter, but not gray matter or CSF.
**Diffusion kurtosis imaging parameters**		
Diffusion kurtosis imaging is a diffusion technique based on the non-Gaussian diffusion of water. It characterizes non-Gaussian diffusion by estimating the excess kurtosis of the displacement distribution and gives an idea of the underlying tissue complexity. We can perform a kurtosis tensor estimation of the water molecule diffusion data like the diffusion tensor estimation technique.	Axial kurtosis	The kurtosis value along the largest eigenvalue direction of the diffusion ellipsoid.
	Mean kurtosis tensor	It quantifies the degree of deviation from Gaussian diffusion and is based only on the trace of the kurtosis tensor.
**Graph analysis parameters**		
If we represent a network as a graph, different mathematical features can be extracted from the graph which represents the topological characteristics of the network.	Bilateral thalamic resting-state network (RSN) degree of symmetry	The percentage of the total voxel number of bilateral thalamic RSNs that correlated with not only the left but also the right thalamus.
	Local efficiency	The local efficiency of a node characterizes how well information is exchanged by its neighbors when this node is removed.
	Modularity	The strength of division of a network into modules.
	Relative betweenness centrality	Betweenness centrality is a measure of the influence of a vertex over the flow of information between every pair of vertices under the assumption that information primarily flows over the shortest paths between them. The betweenness centrality increases with the number of vertices in the network, so a normalized version is often considered with the centrality values scaled between 0 and 1.
	Clustering coefficient	It is a measure of the degree to which nodes in a graph tend to cluster together.
	Minimum spanning tree (MST)	It is a subgraph containing all vertices of the main graph without any loop and with minimum total weights. The total weight of the MST of a sample graph shows the minimal broadcast cost of the main graph.
	Average shortest path	It is also called characteristic path length, is a measure of global connectivity, and shows the efficiency of information exchange in a network. The lesser values show a higher efficiency.
**ERP components**		
Every ERP signal is characterized by peaks and troughs after stimulation time, which are called the ERP components. Although some components have specific names, others are named based on their polarity (P for positive peaks and N for negative ones) and the time of their appearance after stimulation (in millisecond). Sometimes, instead of their appearance time, their appearance order is stated	P300	A positive peak being observed around 300 ms after stimulus time and is mainly related to the consciously controlled attention.
	N350	A negative peak being observed around 350 ms after stimulus onset and is mainly related to cognitive processing during sleep onset period.
	N1	A negative peak being observed around 150–200 ms after the stimulus and is mainly related to auditory attention.
	N2b	A subcomponent of N2 occurring around 200–350 ms post-stimulus and is related to pre-attentive processing in intentional conscious attention.
	Mismatch negativity	A negative peak around 150–250 ms post-stimulus which is related to pre-attentive processing independent of conscious attention.

In general, out of the above-mentioned 66 papers, 42 (63.63%) reported positive results, 45 (68.18%) performed a cross-sectional study, 20 (30.30%) performed a longitudinal study, and one (1.51%) performed both kinds of analysis. Among the total of 46 cross-sectional studies, 29 (63.04%) reported positive results, while among the total of 21 longitudinal studies, 14 (66.67%) also reported positive results. Considering all these studies, a total number of 4,734 subjects (including controls) underwent neuroimaging, with the majority (2,026 participants) being studied using DWI. The main findings are summarized below based on the imaging technique, and more extensive results are presented in Table 1.

### Conventional MRI

Among the 10 studies retrieved (655 participants), three showed positive results (30%), six (60%) were performed cross-sectionally (two with positive results), and four (40%) were performed longitudinally (one with positive results). The main results consist of a smaller volume of cingulate gyrus isthmus, ventromedial prefrontal cortex (vmPFC), and fusiform gyrus.

### Diffusion-Weighted Imaging

Among 24 studies retrieved (2,026 participants), 13 showed positive results (54.17%), 16 (66.67%) were performed cross-sectionally (seven with positive results), and eight (33.3%) were performed longitudinally (six with positive results). The main results consist of alterations of anisotropy and diffusion measures mainly in the corpus callosum, longitudinal fasciculi, and tracts in the internal capsule.

### Functional MRI

**Figure 3:**
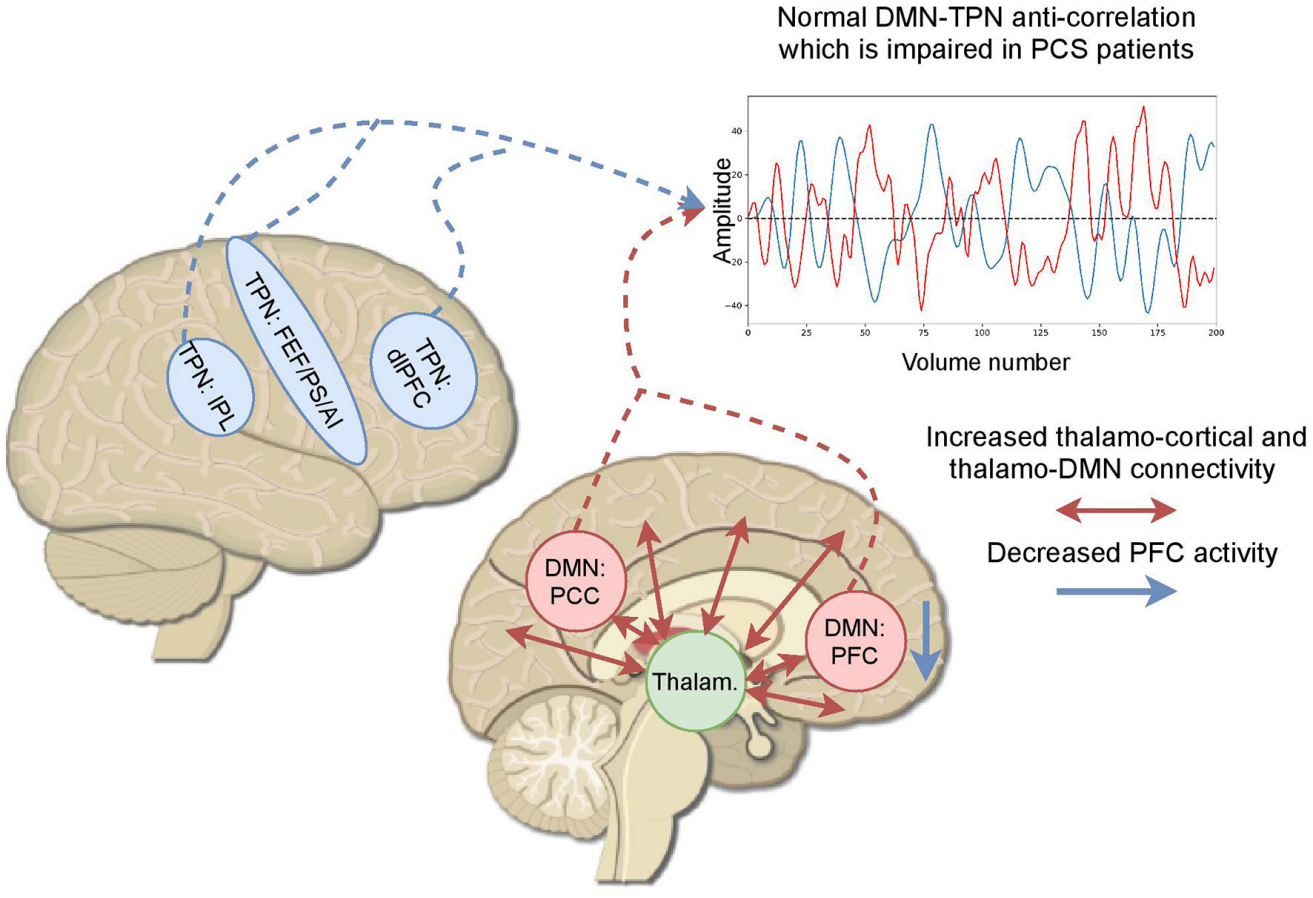
Based on the fMRI studies exploring post-concussive symptoms (PCS), the main findings can be summarized as the abnormal increase of thalamo-cortical connectivity, alterations in the anticorrelation of the default mode network and the task positive network, and a reduction of the activity in the prefrontal cortex. A time series shows a created example of a normal DMN–TPN anticorrelation which becomes altered in patients with PCS. AI, anterior insula; dlPFC, dorsolateral prefrontal cortex; DMN, default mode network; FEF, frontal eye fields; IPL, inferior parietal lobe; PCC, posterior cingulate cortex; PFC, prefrontal cortex; PS, precentral sulcus; TPN, task-positive network.

Among 23 studies retrieved (1,342 participants), 19 showed positive results (82.61%), 14 (60.87%) were performed cross-sectionally (12 with positive results), while eight (34.78%) were performed longitudinally (six with positive results), and one (4.35%) was performed in both types. Seventeen (73.91%) studies used resting-state paradigm for data acquisition, four (17.39%) used cognitive tasks, and two (8.70%) used both paradigms. Out of 19 resting-state studies, 16 (84.21%) reported positive results, and out of six task-based studies, five (83.33%) also reported positive results. The main results consist of alterations of the anti-correlation between the default mode network and the task postive network, which is present in healthy subjects, and an abnormal increase of thalamo-cortical connectivity (see Figure 3).

### Electro(magneto)encephalography

Among nine studies retrieved (five EEG and four MEG; 711 participants), seven showed positive results (three EEG and four MEG; 77.78%), and all nine studies (100%) were performed cross-sectionally (seven with positive results). The main results consist of a reduction of event-related potentials (ERP) components, such as P300, N350, N2b, and N1, and mismatch negativity, an increase of relative theta power, a decrease of relative alpha power, a decrease of beta band inter-hemispheric coherency, and an abnormal slow-wave activity in orbito-frontal cortex, vmPFC, and fusiform gyrus.

## Discussion

This article aims to review the current state of the art in neuroimaging and neurophysiological techniques for the diagnosis of PCS. Out of 80 papers studied in this scoping review, the majority (66 articles) were performed using structural/functional MRI and EEG/MEG, which were conducted either cross-sectionally or longitudinally. In this review, we found that the percentage of positive results is almost the same for both types of study design. However, we should note that the number of cross-sectional studies was greater than twice the number of the longitudinal ones (46 articles vs. 21 articles). Therefore, in order to have a wider overview of the progress of PCS and the biomarkers which can correlate with it more longitudinal studies are needed. Considering the imaging modality, most positive findings were found among studies using functional approaches (i.e., fMRI and EEG/MEG). However, the number of studies using electrophysiological techniques (i.e., EEG) remains limited compared to neuroimaging studies (i.e., fMRI), while the former is more practical and more available in clinical practice. The main findings per technique are discussed below respectively for conventional MRI, DWI, fMRI, and EEG/MEG.

### Conventional MRI and DWI

Considering both conventional MRI and DWI studies, only 47% of the structural MRI studies identified specific abnormalities in patients with PCS. Although DWI studies showed more positive findings compared to conventional MRI (54 vs. 30%), they still have low positive findings compared to functional MRI acquisitions (54 vs. 83%). However, we should notice that, in clinical practices, the acquisition of good-quality DWI images is much harder than acquiring good-quality MRI due to the high sensitivity of DWI images to motion. Structural MRI allows one to evaluate anatomical and structural brain damage in a qualitative or quantitative way using different acquisition sequences—for example, a conventional T1-weighted sequence is suitable to quantify and analyze changes in the volume and thickness of different brain regions. It is known that brain volume loss (whether globally or regionally) is a consequence of moderate or severe brain injuries (16)—for example, the anterior cingulate and left cingulate gyrus isthmus in the white matter are among the regions which show atrophy over time after a brain injury (16). Because the anterior parts of the cingulum play vital roles in different cognitive systems [e.g., working memory; (77)], atrophy in this area can explain the persistent symptoms after the concussion. The gray matter volume within vmPFC and the fusiform gyrus was also found to positively correlate with PCS symptoms, specifically emotional functioning (18). This may be due to the fact that the vmPFC has a strong connection with the amygdala and is involved in self-control and decision making (78).

A widely used MRI sequence to study the structural changes of the brain after PCS is DWI, which is mainly used to study changes of the white matter tracts. To analyze these tracts quantitatively, four main parameters of fractional anisotropy, axial diffusivity, radial diffusivity, and mean diffusivity were explored. The PCS-associated white matter abnormalities can be mainly characterized by a decrease in FA values and an increase in MD, AD, and RD values, respectively, in the injured tracts (FA and MD were the most commonly reported parameters to be altered compared to the others). The main reported tracts with axonal injury in PCS patients were the corpus callosum (24–26, 28, 34, 47), the longitudinal fasciculi (24–26, 34, 42), and tracts in the internal capsule (24, 26, 28, 34, 47). As these tracts are the main pathways of widespread cortical and subcortical connections, any axonal damage in these tracts can lead to various cognitive symptoms in patients with PCS. These axonal damages are mainly due to the rotational acceleration and/or deceleration forces happening when the concussion occurs (24). In addition to these main tracts, altered measures of anisotropy and diffusivity were also reported in the fronto-occipital fasciculi (24, 25, 47), the uncinate fasciculus (24, 41, 42), the cortico-spinal tract (25), the thalamic radiation (25, 28, 34, 42), the cingulum bundle (28), and the pontine tegmentum (36). By applying tractography algorithms on the DWI images, structural connectivity can also be analyzed between different cortical regions. Using this technique, it has been shown that, compared to patients with post-traumatic stress disorder (PTSD) only, subjects with PCS and PTSD have a greater diversity in their structural connectivity between the hippocampus and the striatum, which could be associated with PCS but not PTSD (44).

In summary, the structural neuroimaging studies evaluating gray matter and white matter integrity found impairments of the cingulate gyrus and the prefrontal area, while for the white matter the corpus callosum, the longitudinal fasciculi, and internal capsule were the most frequently reported structures to be impaired or linked to PCS severity. As we can see and also highlighted by Miller et al. (37), the axonal damages in patients with PCS are spatially heterogeneous. This could be due to the heterogeneity of the studied populations (athletes vs. regular people vs. veterans) and the heterogeneity of the symptoms (e.g., stress, headache, sleep disturbances, and attentional issues).

### Functional MRI

In fMRI studies, the results can be presented based on changes in BOLD activation level or based on functional connectivity among regions (i.e., network-based analysis). Furthermore, functional networks can be summarized by graph analysis metrics which create a mathematical framework to study brain network topology in normal and pathological states in more detail. We discuss the fMRI findings of PCS under these different categories.

#### BOLD Activation Level Analysis

Among the studies using this technique, most of them looked at working memory and selective attention deficits, which are the two main cognitive domains affected after mTBI (48). Based on previous studies, the brain areas which are involved in these cognitive domains are mainly the dorsolateral and the ventrolateral prefrontal cortex, the supplementary motor area, the premotor area, posterior parietal area, and the anterior cingulate cortex (48, 77, 79). The activation level of these areas during tasks requiring a high cognitive load is correlated with PCS severity, showing increased attentional and short-term memory demands (48). Some studies also showed hyperactivation in the parahippocampal gyrus and the posterior cingulate cortex (PCC), which are mainly related to episodic memory and memory retrieval (48). The activation of these regions in high-load working memory and selective attention tasks shows a potential cerebral compensatory response to a possible microstructural injury in patients with PCS (48). Hyperactivity was also observed in the left angular gyrus, which resulted in the same behavioral accuracy for patients with PCS and healthy subjects in performing a working memory task (69). Taking all together, it can be stated that patients with PCS use more cognitive resources to perform an attention or working memory task with the same accuracy as the healthy subjects.

A decrease in BOLD activation level in the medial prefrontal cortex was also observed in patients with PCS (50, 61). It is known that this region is important for executive functioning and emotion regulation (80, 81). In addition, it is a core area of the default mode network (DMN) and an important relay station between DMN and other executive networks (61). As a result, impairment in this area can lead to serious cognitive and affective impairments (50, 61). Using transcranial magnetic stimulation as a treatment, an increase of the medial prefrontal cortex activation level during a working memory task was found and was associated with less severe PCS symptoms (60).

Besides working memory and selective attention, light sensitivity and headaches were also studied using a visual tracking task. A higher BOLD activity in the middle temporal/lateral occipital regions was observed compared to healthy subjects when the patients were exposed to visual stimuli. These regions are known as extra-striate visual regions involved in motion and object processing. The higher activation in these regions was also associated with higher FA values in the same region. In sum, light sensitivity and the associated headaches could be related to an abnormal sensitivity of motion-sensitive neurons (40).

#### Functional Connectivity Analysis

In a general view, abnormal functional connectivity can be found in almost all the functional networks which can be detected in the resting state (51). While few studies have reported an abnormal whole-brain hyperconnectivity especially in the acute phase (65), other studies have shown an overall whole-brain connectivity reduction in these patients (56, 63). Whole-brain connectivity analysis gives us a general view of the functional organization of the brain; however, focusing on specific networks or regions, chosen in a hypothesis-driven way, gives more insights about network configuration changes and a more fine-grained understanding of the pathology. Among different networks and regions, DMN and the thalamus were found to be the most affected ones.

##### Default Mode Network

The DMN is a network mainly related to internal awareness (82). Posterior regions of the DMN, such as the precuneus, showed more vulnerability to traumatic injury (53). A decrease of the connectivity of the precuneus inside the DMN and its increased connectivity with the primary and secondary visual networks and the left fronto-parietal network can alter the transition of attention from internal to external awareness (53). Another critical area in the DMN is the PCC. The decreased connectivity of the PCC within the DMN is associated with an increased connectivity of the medial prefrontal cortex (mPFC), which potentially shows the compensatory role of the mPFC to the impaired neurocognitive functions. Indeed the mPFC and the PCC are intrinsically interdependent inside the DMN, and their function is highly complementary (53). The anterior regions of the DMN, such as the anterior cingulate cortex (ACC), have also shown altered connectivity in these patients. The connectivity of the ACC with the emotional, the primary visual, and the higher-order processing networks is negatively correlated with the severity of the PCS (51). Considering the role of the ACC in pain processing, error and conflict detection, behavioral and cognitive control, as well as emotional processing, this could potentially explain the different complaints in mTBI patients. In addition to the role of the DMN in internal awareness, the interaction between the DMN and the task positive network (TPN) is crucial for performing executive functions. In fact, the balance between internal and external awareness is supported by the interaction of these two networks with temporally alternating higher activity states (83). In patients with PCS, the deactivation of the DMN is not strong enough while they are performing a working memory task (61). Specifically, an increase in the connectivity between the DMN and the TPN has been observed (58, 61, 68). This altered anti-correlation between the DMN and the TPN (i.e., the lack of silencing of the DMN) could explain some of the difficulties in task performance that patients with PCS suffer from.

##### Thalamus

This subcortical hub plays an important role in memory, executive functions, attention, and emotion among others (81). Different studies have shown a PCS-related increase of the thalamo-cortical connectivity (49, 58, 64) which can lead to an inefficient neuronal activity (64). The increased thalamo-cortical connectivity can be related to the alteration of the GABAergic inhibitory neurons in the thalamus (49, 58), resulting in a reduction of inhibitory control over the thalamo-cortical activity. The connectivity of the thalamus with other networks has also been studied. Evidence especially suggests that the connectivity between the thalamus and the DMN increases in patients with PCS (58, 62). As mentioned, a well-coordinated interaction between the DMN and the TPN is necessary for an appropriate balance between internal and external awareness and proper task execution. The fronto-parietal control (FPC) network (62) and the salience network (84) were suggested as networks that control this balance between the DMN and the TPN. The thalamus has connections with all of these networks and has a key role in this complex inter-network interaction. An increased connectivity between the thalamus and the DMN could lead to a malfunctioning of this modulatory interaction. In addition to the increased connectivity of both the thalamus and the DMN, a decrease of connectivity between the thalamus, the FPC, and the DAN was also reported (62). A normalization of this decreased connectivity was correlated with better PCS scores, while the connectivity with the DMN remained unchanged.

##### Other Regions

Besides the DMN and the thalamus, the reduction of homotopic functional connectivity in the pain processing regions is another finding which was shown to be normalized with symptom recovery and can serve as another potential biomarker of PCS (66).

#### Graph Analysis

One approach to study brain networks and their topological characteristics is the data-driven graph analysis. In this approach, connectivity measures are represented by weighted graphs [nodes represent regions of interest (ROIs), and edges represent connectivity between ROIs]—for example, graph modularity is increased in the temporal regions in the subacute phase and is decreased in the frontal regions in the chronic stage in patients with PCS (54). These changes can be localized mainly in regions related to executive functions, working memory, cognitive control, and attention. Some mathematical features of the graph (detailed in Table 2), such as the clustering coefficient, are also correlated with increased thalamo-cortical connectivity (64). Combining the graph analysis results of the functional networks with the diffusion-weighted images, it was revealed that regions of the brain showing a lower local relative betweenness centrality in the functional network have also lower fractional anisotropy; in addition, a decrease of global efficiency of the functional networks is related to diffuse axonal injury (64). The average shortest path weights and minimum spanning tree weights have also been shown to be increased in patients with PCS (67). Taking all these results together, the graph analysis shows that patients with PCS tend to have costly and less efficient network configurations for information transfer (67), which lead to cognitive impairments.

To summarize, the fact that positive findings were found for 82% of the papers showed that impairment in functional connectivity after mTBI could potentially explain most of the post-concussive symptoms. Mainly, besides a whole-brain connectivity reduction, alterations of the connectivity in the DMN and its interaction with other executive networks, especially alterations of the anti-correlation between the DMN and the TPN, could be associated with cognitive impairments. In addition, an increase of the thalamo-cortical connectivity due to alteration of GABAergic inhibitory neurons in the thalamus can also lead to difficulties in task performance. These functional impairments may recruit compensatory mechanisms in the brain and use extra cognitive resources while cognitive tasks are performed.

### Electro(magneto)encephalography

In general, the EEG/MEG data can be analyzed whether in the sensor space (signals directly from surface sensors) or in the source space (i.e., signals of surface sensors are projected to the cortex to estimate the cortical location of the neural generators). Furthermore, various analysis methods can be performed on the standard frequency bands of the signals: delta (0–4 Hz), theta (4–8 Hz), alpha (8–12 Hz), beta (12–30 Hz), and gamma (>30 Hz). In addition, the EEG/MEG recordings can be done either in resting state or in response to stimuli, averaged over many trials, to measure ERP in case of EEG recording or event-related fields in case of MEG recording. Although in the analysis methods there are no serious differences between EEG and MEG signals in terms of clinical applicability and data acquisition, they are completely different. While EEG is widely available and can be performed easily in different clinical settings, MEG is scarcely available for clinical purposes. In the present review, eight out of 10 EEG/MEG studies identified neurophysiological changes linked to PCS (six EEG studies with four reporting positive results and four MEG studies all reporting positive results).

Considering ERP studies, they were mainly focused on the amplitude of P300 (69, 76), N350 (50, 69), N2b, N1, and mismatch negativity (MMN) components (76). All of them reported a reduction in the amplitude of such components. The first ERP study found that the amplitude of the N350 component in the frontal electrodes was decreased in patients with PCS while performing a working memory task compared to a control task (50, 69). Because this N350 amplitude reduction was correlated to the lower BOLD signal changes of the right mid-dorsolateral prefrontal cortex (mid-DLPFC) in the same task, it is thought that this specific ERP component is related to the neural activity of the mid-DLPFC. In addition, the amplitude of P300 in parietal regions is decreased after a brain injury, and this reduction in amplitude is more significant when patients with PCS suffer from severe depressive symptoms (69). While both N2b and MMN are related to pre-attentive processing, MMN is observed independent of conscious attention, and N2b is observed in intentional conscious attentions (76). As a result, the reduction of amplitude of P300, N2b, and MMN can be linked to the attentional deficits which are common in PCS. The N1 component mainly reflects auditory attention and shows a reduced amplitude in case of auditory processing difficulties (76). In the analysis of resting-state EEG data, increase of relative theta power, decrease of relative alpha power, and decrease of beta band inter-hemispheric coherence have been observed (75).

Considering MEG recordings, it has been shown that the total MEG magnitude in the source space is correlated with the PCS scores (71). Slow-wave features are still more informative for cognitive impairment examination in this cohort of patients (72)—for example, the abnormal slow-wave activity in the orbitofrontal cortex and vmPFC has been shown to be associated with personality changes, concentration troubles, and emotional instability (71). Because these regions are highly connected to other cortical and subcortical areas, this abnormal activity can cause impairments in a vast domain of cognitive abilities (71). In addition, visual difficulties, such as blurred vision, have been explained by the abnormal slow-wave activity in the right fusiform gyrus (71), an important region for face, object, and body recognition and processing (85). The abnormal slow-wave generation in different regions has been associated with white matter abnormalities or micro-structure damages to the major tract in the same regions (86). The connectivity analysis features of MEG data in different frequency bands have also been shown to be informative biomarkers to study PCS after mTBI. Inter-regional resting-state phase synchrony in the alpha band could classify patients from healthy subjects with an accuracy of 80%, in which the distance of each participant to the classification boundary was correlated with the symptom severity of that subject (73). The functional connectivity of ROIs in the prefrontal cortex, medial temporal lobe, putamen, and cerebellum with the whole brain was increased, while the functional connectivity of the right frontal pole with the rest of the brain in patients with PCS was decreased (74). These abnormal connectivity variations were observed in the beta, gamma, and low-frequency bands (i.e., 1–7 Hz). The decrease of functional connectivity in the right frontal pole can be explained by diffuse axonal injury, which leads to the disruption of neural communications (87). On the other hand, the increase of functional connectivity in the prefrontal cortex, the medial temporal lobe, the putamen, and the cerebellum could be explained by the overexcitation of glutamate in the pyramidal neurons of the cortex, which may lead to an alteration of GABAergic interneurons (74).

Taking all these information together, different alterations in the EEG/MEG markers may be related to the white matter abnormalities and axonal injury, alterations of GABAergic neurons, and functional dysfunction of frontal regions, such as the DLPFC.

### Limitations

Our work has some limitations that need to be taken into account. First of all, the heterogeneity of the studied populations resulted in the heterogeneity of the symptoms and the imaging markers. Secondly, a few number of studies explored PCS longitudinally. These two limitations made us unable to have specific strong prognostic recommendations for PCS. The third limitation was the few number of studies which used imaging techniques other than MRI and EEG. While imaging techniques like PET, SPECT, and fNIRS can provide more insight about the brain alterations in PCS. The few number of studies limited the generalization of their findings and therefore, were excluded from the analysis. Finally, most of the studies that we included in our review did not have a specific hypothesis, and the questions they were trying to answer were really diverse. This led to two main limitations of this review. First, we could not perform a meta-analysis which could help us to understand PCS more deeply. Second, because in the exploratory studies the number of biomarkers that the authors were planning to study is not known *a priori*, a comparison between the number of tested markers and the number of positive findings could not be performed in an appropriate and accurate way.

## Concluding Remarks

In this scoping review, we studied the most reliable approaches to evaluate PCS and the common findings among studies which could explain PCS (i.e., biomarkers of PCS). Most of the studies using structural MRI to investigate the neural correlates of PCS did not find any specificities, except for an atrophy mainly involving the cingulate and the prefrontal areas linked to PCS symptoms, and abnormalities in the anisotropy and diffusion parameters of the corpus callosum, the longitudinal fasciculi, and the internal capsule. On the other hand, the majority of functional studies (fMRI and EEG/MEG) found an abnormal increase in the thalamo-cortical functional connectivity, which could be linked to the employment of compensatory mechanisms needed to perform cognitive tasks. In addition, an altered anti-correlation between the DMN and the TPN could explain some of the difficulties in task performance experienced by patients with PCS. Besides this, reduction of brain activity in specific areas was also found; the most frequently reported ones being the prefrontal cortex. The functional abnormalities in these above-mentioned brain regions and networks should draw the attention of physicians to provide appropriate care to these at-risk patients. The EEG/ERP studies identified a reduction in ERP component amplitudes related to attentional processes. However, only six studies used EEG, although compared to MRI or even MEG, this technique is easier to use in clinical practice. Further EEG studies could provide an insightful prediction of PCS outcome that could be more easily implemented in clinical practice. In addition, considering the heterogeneity of the results based on different neuroimaging modalities, it is recommended to conduct more multimodal studies. Furthermore, to decrease the amount of inconsistency of the results across studies, more longitudinal multi-center studies with bigger sample sizes are advised to be performed. It should be noted that, in most of the studies included in the review, no *a priori* or precise hypotheses were stated. We hope that our findings will guide authors to postulate specific hypotheses for future neuroimaging studies. So far, fMRI seems the most robust approach to study PCS severity, and future prospective longitudinal studies in a large sample of concussed patients using clinically relevant techniques such as EEG should determine the prognostic factors of good and poor outcome following a concussion.

## Data Availability Statement

The original contributions presented in the study are included in the article/Supplementary Material, further inquiries can be directed to the corresponding authors.

## Author Contributions

SM and MMF: conceptualization, literature search, and writing. MAFC: literature search. AT: conceptualization, writing, and supervision. JA, J-FK, NL, GM, and SL: writing—review and editing. All authors gave final approval of the manuscript.

## Funding

This study was supported by the University and the University Hospital of Liege, the Belgian National Funds for Scientific Research (FRS-FNRS), the European Union's Horizon 2020 Framework Programme for Research and Innovation under Specific Grant Agreement No. 945539 (Human Brain Project SGA3) and No. 686764 (Luminous project), DOCMA project (EU-H2020-MSCA-RISE-778234), the European Space Agency (ESA) and the Belgian Federal Science Policy Office (BELSPO) in the framework of the PRODEX Programme, Fondazione Europea di Ricerca Biomedica, the Bial Foundation, the Mind Science Foundation and the European Commission, the fund Generet, the Mind Care International Foundation, and the King Baudouin Foundation.

## Conflict of Interest

The authors declare that the research was conducted in the absence of any commercial or financial relationships that could be construed as a potential conflict of interest.

## Publisher's Note

All claims expressed in this article are solely those of the authors and do not necessarily represent those of their affiliated organizations, or those of the publisher, the editors and the reviewers. Any product that may be evaluated in this article, or claim that may be made by its manufacturer, is not guaranteed or endorsed by the publisher.

## Abbreviations

ACC: anterior cingulate cortex
MKT: mean kurtosis tensor
AD: axial diffusitivity
MMN: mismatch negativity
AI: axonal injury
MPFC: medial prefrontal cortex
BC-PSI: British Columbia Postconcussion Symptom Inventory
MRS: magnetic resonance spectroscopy
BOLD: blood-oxygen-level dependent
mTBI: mild traumatic brain injury
CT: computed tomography
OFC: orbitofrontal cortex
DAN: dorsal attentional network
PCC: posterior cingulate cortex
DMN: default mode network
PCD: post-concussion disorder
DLPFC: dorsolateral prefrontal cortex
PCS: post-concussion syndrome
DSM-IV: Diagnostic and Statistical Manual for Mental Disorders—IVth Edition
PCS: post-concussion syndrome negative
DTI: diffusion tensor imaging
PCS+: post-concussion syndrome positive
DWI: diffusion-weighted imaging
PCS-NIM: PCS—negative impression management
EEG: electroencephalography
PCSQ: Post-concussive Symptom Questionnaire
ERF: event-related field
PET: positron emission tomography
ERP: event-related potential
PO: poor outcome
FA: fractional anisotropy
PTSD: post-traumatic stress disorder
FC: functional connectivity
RD: radial diffusitivity
FLAIR: fluid-attenuation inversion recovery
ROI: region of interest
fMRI: functional magnetic resonance imaging
RPQ: Rivermead Post-concussion Symptoms Questionnaire
fNIRS: functional near-infrared spectroscopy
RSN: resting-state network
FPC: fronto-parietal cortex
SCAT: Sport Concussion Assessment Tool
GM: gray matter
SN: salience network
GO: good outcome
SP: shortest path
ICBs: intracranial bleeds
SPECT: single photon emission computed tomography
ICD-10: International Classification of Diseases−10th Edition
SWI: susceptibility-weighted imaging
IFO: inferior fronto-occipital fasciculus
TBI: traumatic brain injury
ImPACT: immediate post-concussion assessment and cognitive testing
TPN: task positive network
MD: mean diffusitivity
vmPFC: ventromedial prefrontal cortex
MEG: magnetoencephalography
WM: white matter.
